# Electrospun Parallel, Crossed Fibers for Promoting Cell Adhesion and Migration

**DOI:** 10.3390/ma18143224

**Published:** 2025-07-08

**Authors:** Xiang Gao, Jingjun Peng, Linjie Huang, Xiaoquan Peng, Yanjun Cheng, Wei Zhang, Wei Jia

**Affiliations:** 1 National Innovation Center for Advanced Medical Devices, National Institute of Advanced Medical Devices, Shenzhen 518110, China; gao_xiang0704@163.com (X.G.); jj.peng@nmed.org.cn (J.P.); lj.huang1@nmed.org.cn (L.H.); xq.peng@nmed.org.cn (X.P.); chengzideguai@hotmail.com (Y.C.); 2 Centre for Translational Medicine Research and Development, Shenzhen Institute of Advanced Technology, Chinese Academy of Sciences, Shenzhen 518055, China

**Keywords:** electrospun fiber, cell behavior, human skin fibroblasts

## Abstract

Electrospun fibers, possessing biomimetic characteristics similar to fibrous extracellular matrices, have attracted widespread attention as scaffold materials for skin tissue engineering. The topographical structure of electrospun fibers plays a critical role in determining cell behavior. However, the effects of fiber topography on human skin fibroblasts (HSFs) remain unclear. In this study, electrospinning technology was employed to investigate how parallel and crossed fiber architectures influence the spreading morphology, proliferation, and migration of HSFs. The results demonstrated that cells exhibited spindle-shaped elongation along single fibers; on closely spaced parallel fibers, cells formed cross-adhesions between adjacent fibers, with a fiber spacing of 30–60 μm serving as the threshold range for distinguishing individual cell behaviors. At fiber intersections, a characteristic spacing of 100 μm distinguished three distinct cellular responses: anchoring, turning, and bridging. The probability of a cell altering its preexisting migration path depended on its ability to extend laterally and reach adjacent fibers, which was constrained by the upper limit of the cell body’s minor axis. This study elucidated the unique role of the electrospun fiber topography in guiding cellular decision-making in complex microenvironments, provided important insights into topography-triggered cell migration, and highlighted the practical significance of material-guided strategies in tissue engineering.

## 1. Introduction

Skin is the largest organ in the human body, composed of collagen fibers and elastic fibers [1]. Collagen fibers provide mechanical stability, elasticity, and strength to the tissue, while elastic fibers often exhibit a wavy pattern, allowing them to return to their original state after stretching [2]. In the dermis, the organization of collagen fibers is dynamic and varies regionally to meet the functional demands of different skin areas. Near the skin surface, particularly along Langer’s lines [3], collagen fibers are predominantly arranged in a relatively parallel manner, with variable spacing between fibers. This arrangement enables the skin to accommodate external stretching forces. In contrast, within the reticular and papillary layers of the dermis, collagen fibers adopt a web-like cross-linked structure, which supports the attachment between the epidermis and dermis and enhances the skin’ s mechanical resilience [4]. In the skin repair phase, human skin fibroblasts (HSFs) within the extracellular matrix (ECM) play crucial roles in tissue synthesis and remodeling. These HSFs undergo proliferation, migrate toward the wound site, and activate into myofibroblasts [5,6]. Through the secretion of enzymes and proteins, they establish a highly organized cellular scaffold that facilitates contraction and wound healing, which is essential for matrix synthesis and repair [7,8]. Notably, the topology of collagen fibers directly governs the polarity distribution, migratory trajectories, and phenotypic transitions of HSFs via mechanotransduction signaling. Consequently, understanding how collagen fibers modulate the behavior of HSFs is of paramount importance [9,10,11,12].

Electrospinning, a fiber fabrication technique utilizing various synthetic or natural polymers, is employed to create fibers that mimic the morphological characteristics of the ECM [13]. Adjusting electrospinning parameters such as the injection speed, voltage, and collector type can modify fiber properties including the diameter, surface morphology, and fiber orientation, enhancing the emulation of the ECM structure [14]. Consequently, electrospinning has found broad applications in the biomedical field [15]. Oriented fibers, characterized by their simplified structure, have attracted increasing attention for facilitating a clearer interpretation of interactions between cells and scaffold materials [16]. Recent research has shown that aligned fibrous structures significantly enhance cellular adhesion, directional migration, and extracellular matrix synthesis when compared to randomly distributed fiber networks [17,18]. Shao et al. [19] proposed that well-aligned fibers, structurally resembling natural collagen fiber bundles, hold potential as scaffolds for bone regeneration. However, current research has predominantly focused on electrospun membranes, where numerous influencing factors (such as fiber entanglement and the mechanical or structural properties of the underlying substrate) complicate the study of cell–matrix interactions. There has been a shortage of reports examining the impact of the fiber topological structure on cell behavior while excluding various factors. Additionally, current research has predominantly emphasized static observations based on in vitro cell culture [20]. Conversely, dynamic interactions between cells and biomaterials are paramount, as they govern cell functions such as adhesion, spreading, migration, and differentiation, ultimately influencing tissue formation. Hence, it is essential to investigate the dynamic behavior of cells on the surface of the fiber scaffold surface.

To emulate the structure of the natural collagen fibers found in the dermal layer of the skin, this study employed the electrospinning technique to blend semi-crystalline polymer Polyurethane (PU) with biocompatible Poly (lactide-co-caprolactone) (PLCL). The addition of the thickening agent Polyethylene oxide (PEO) facilitated the preparation of single-layered oriented and grid-like fibers [21]. A subsequent observation of cell behavior aimed to meticulously uncover the influence of distinct matrix cues on cellular responses, offering valuable insights and innovative strategies for biomaterial design and fabrication.

## 2. Materials and Methods

### 2.1. Materials

Hexafluoroisopropanol (HFIP) was obtained from Shanghai Macklin Biochemical Co., Ltd. (Shanghai, China). Agarose was purchased from Sangon Biotech (Shanghai) Co., Ltd. (Shanghai, China). PLCL (LA/CL = 70/30, 200 kDa) was supplied by Yongkang Leyi Co., Ltd. (Beijing, China). PU was purchased from Sigma Aldrich Trading Co., Ltd. (Shanghai, China). PEO was supplied by Shanghai Macklin Biochemical Co., Ltd. (Shanghai, China). Fetal bovine serum (FBS) was obtained from Gibco (Grand Island, NY, USA). The phalloidin-iFluor 488 assay kit was obtained from Abcam (Cambridge, MA, UK). Phosphate buffer saline (PBS) solution was purchased from Sigma-Aldrich (St. Louis, MO, USA).

### 2.2. Fabrication of Micro-Scale Fiber

**Figure 1:**
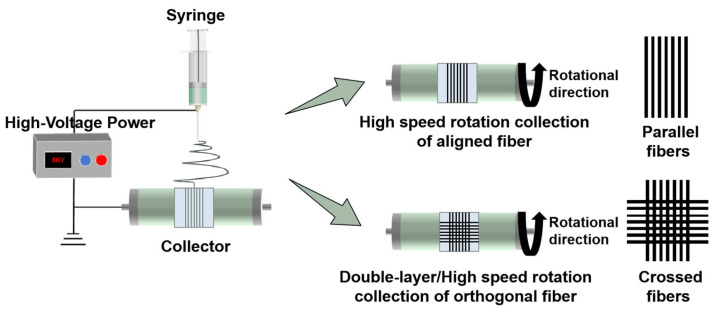
Preparation process of electrospun fibers with different topologies.

The micro-scale fiber was composed of PU and PLCL in a 1:1 proportion [22,23], with the addition of 3% PEO to the mixing solution. A polymer solution was prepared by dissolving predetermined amounts of PU and PLCL in hexafluoroisopropanol (HFIP) at a final concentration of 70 mg/mL. The mixture was stirred magnetically at an ambient temperature for 15 h to ensure complete dissolution. Electrospinning was performed using a high-voltage electrospinning machine (Tianjin Yunfan Technology Co., Ltd., Tianjin, China). The solution was loaded into a plastic syringe equipped with a 19-gauge stainless steel needle. The main parameters of electrospinning are presented in Table 1. Fiber deposition was accomplished using cover glasses coated beforehand with a 2% agarose solution and subsequently dried in an oven, to obtain a “background non-adhesive” fiber-patterned deposition platform. These cover glasses were then adhered to aluminum foil and secured onto a rotary collector. The fabrication of crossed fibers necessitated a 90-degree rotation of the aluminum foil after the production of parallel fibers, followed by the collection of fibers in an orthogonal direction. For parallel fibers, the deposition time was 200 s. For crossed fibers, fibers were deposited in two orthogonal directions, with each direction electrospun for 200 s, respectively. The preparation process is shown in Figure 1. To evaluate the influence of fiber orientation on cell behavior, randomly oriented fibers were additionally fabricated as a control group alongside parallel and crossed fiber scaffolds. Detailed fabrication procedures are described in the Supplementary Material.

### 2.3. Surface Morphology

The morphology and structure of the electrospun fibers were visualized using an optical microscope (Olympus, Tokyo, Japan). All fiber diameters and distribution angles were measured using ImageJ 1.53c software.

### 2.4. Chemical Structure

Fourier transform infrared spectroscopy (FTIR, Bruker Alpha, Bruker, Ettlingen, Germany) was employed to characterize the chemical bonding profiles of the electrospun membranes. The infrared spectra of both the fabricated membranes and raw PEO powder were recorded in attenuated total reflectance (ATR) mode over a wavenumber range of 400–4000 cm^−1^.

### 2.5. Cell Compatibility

Human skin fibroblasts (HSFs, KCB2012087YJ) were procured from the Kunming Cell Bank, Chinese Academy of Sciences (Kunming, China). Cells were maintained in high-glucose Dulbecco’s Modified Eagle’s Medium (DMEM), enriched with 10% fetal bovine serum (FBS) and 1% penicillin–streptomycin, and incubated in standard conditions (37 °C, 5% CO_2_). The culture medium was replaced every two days. Once HSFs reached approximately 90% confluence, and during passages 3 to 6, they were harvested using trypsin digestion and seeded onto pre-sterilized electrospun scaffolds for subsequent experimentation.

The fiber structures were fixed in a six-well plate (one structure per well), disinfected with an ethanol solution overnight, and then thoroughly washed with PBS. Subsequently, 2 mL cell suspensions were seeded per well at a density of 5 × 10^4^ cells/mL. It is important to ensure that during the cleaning and cell implantation processes, the medium remains undisturbed to prevent the disruption of fiber structures or unintended morphological alterations. After three days of cultivation, rinsing three times using PBS, the samples were fixed with glutaraldehyde, then dehydrated with a series of ethanol solutions (30%, 40%, 50%, 70%, 80%, 90%, 95%, 100%), air-dried, and then gold-sprayed before the samples were viewed using a scanning electron microscope (SEM, JSM-7100F, JEOL, Tokyo, Japan).

To assess cellular morphology and proliferation on the electrospun scaffolds, immunofluorescence labeling with phalloidin-iFluor 488 was performed. Cells cultured on the fibrous substrates for 1, 4, and 7 days were fixed in 4% paraformaldehyde for 30 min, followed by three rinses with phosphate-buffered saline (PBS), each lasting 5 min. Permeabilization was achieved using 0.1% Triton X-100. Subsequently, 50 μL of a 1× phalloidin solution was applied to each well and incubated in the dark for 1 h. Cell nuclei were counterstained with DAPI. Confocal laser scanning microscopy (STELLARIS 5, Leica Microsystems, Wetzlar, Germany) was employed for image acquisition [24,25]. To illustrate the morphological characteristics associated with distinct cellular behaviors, cell contours were extracted from fluorescence images using Image J.

As previously described [26], after the cells had been cultured on the fibers for some time, cell quantification was performed using ImageJ software. The cell proliferation rate was determined using the following formula:

$$D=\frac{n_{\mathrm{cell}}}{S}\;\;\;\;P=\frac{D(t_{2})-D(t_{1})}{D(t_{1})}\times 100\%\;(t_{2}>t_{1})$$

where *n*_cell_ refers to the total number of cells, *S* represents the total surface area of the fibers, *P* denotes the cell proliferation rate, and *D*(t_1_) and *D*(t_2_) refer to the cell density on the fibers in the initial or end state.

### 2.6. Cell Migration

The prepared fibers underwent a 12 h immersion in 75% ethanol to eliminate the water-soluble component PEO from the fibers [27]. Subsequently, cell seeding was carried out after UV sterilization. Time-lapse imaging was conducted within a live-cell workstation, maintaining a 5% carbon dioxide atmosphere and a temperature of 37 °C. This imaging process extended over a 12 h timeframe. Image J software was employed to compute various parameters related to cell migration [28]. The cell migration speed was determined by dividing cell displacement by time, while the cell migration rate was calculated by dividing the cell path length by time. The effective migration rate was computed as cell displacement divided by the cell path length. Finally, the relationship between fiber spacing and cell behavior was analyzed and counted.

The two-dimensional Laplace law model can be used to describe the correlation between cellular geometry and the mechanical tension transmitted from the fibrous matrix to the cell and to predict changes in intracellular forces within the fiber network [29,30]. In this model, the relationship among the bridging edge radius *R*, the linear tension *λ* along the curved bridging edge, and the two-dimensional membrane tension *σ* (the overall two-dimensional normal tension) was expressed as follows: *R* = *λ*/*σ*. An analysis of the force balance model indicated that the contractile force generated by the cell was positively correlated with the radius *R* [31]. The radius *R* was also influenced by the distance *d* between the two endpoints of the bridging arc, with *d* serving as an indicator of the magnitude of tension. The ratio of *R* to *d* reflected intracellular tension [30]. Based on this model, the bridging edges along the cell periphery were identified to investigate the associated line tension and membrane tension during migration. The length *L* of the actin bridge enriched along the non-adhesive edge and the distance *d* between the arc endpoints can be expressed in terms of the radius *R* of the bridging edge and the chord angle *φ* as follows: *L* = *φR*, *d* = 2*R*sin(*φ*/2). In the two-dimensional Laplace law model, each parameter was independently varied and redefined to assess the sensitivity of the bridging structure to fluctuations in these parameters. The actomyosin contractile force was determined by the actin bridge length *L*, the density of myosin motors *D*_m_, the displacement resistance *ξ* of stress fibers, and the traction force *k* generated by individual myosin molecules. The one-dimensional line tension along the stress fibers and the two-dimensional membrane tension were given by the following: $\lambda =kD_{m}L/(L+\xi )$, $\sigma =kD_{m}L/R(L+\xi )$. By manipulating the above equations, the linear tension along the bridging edge and the membrane tension can be obtained as follows: $\lambda =kD_{m}\{1/(1+\xi {\left[2R\arcsin(d/2R)\right]}^{-1})\}$, $\sigma =kD_{m}\{1/(1+\xi {\left[2R\arcsin(d/2R)\right]}^{-1})\}/R$. Before proceeding with the calculations, parameter values were first assigned based on reference [29], *k* = 1, ξ=1.2, $D_{m}=5R^{1.4}$.

## 3. Results and Discussion

### 3.1. Morphology and Structure of Electrospun Fibers

**Figure 2:**
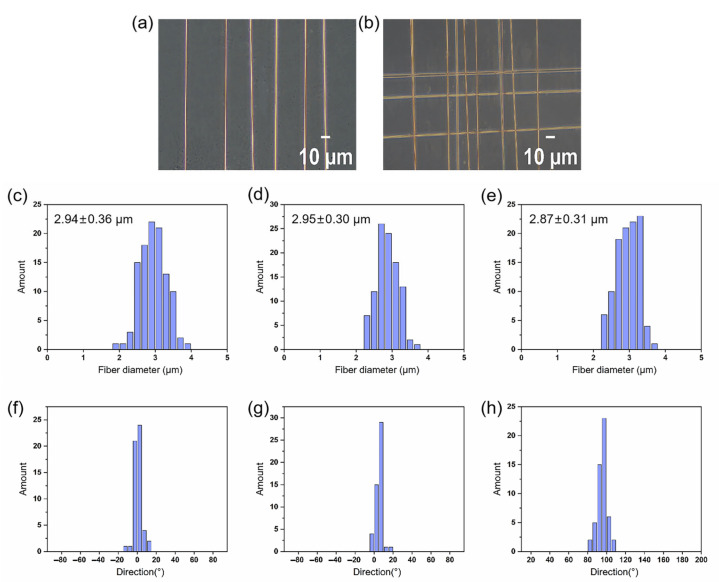
Optical micrograph and analysis results of electrospun fibers with different topological structures. (**a**,**b**) Optical micrograph. (**c**–**e**) Histograms of fiber diameter distribution (n = 106). (**f**–**h**) Histograms of fiber angle distribution (n = 106).

After optimizing the parameters of electrospinning, optical microscopy was used to examine the surface morphology of the electrospun fibers, as depicted in Figure 2a,b. All fibers exhibited smooth and uniform surfaces without bead formation. Parallel fibers were aligned unidirectionally, whereas crossed fibers were arranged along two perpendicular directions. The diameter measurements of fibers from each group are presented in Figure 2c–e. The average diameter of parallel fibers was 2.9 ± 0.4 μm, while the average diameters of crossed fibers along the two directions were 3.0 ± 0.3 μm and 2.9 ± 0.3 μm, respectively. There were no significant differences in the fiber diameter among the groups. The selection of this diameter was guided by our observation that aligned fibers with a diameter of 3 μm provided both sufficient mechanical integrity and effective alignment control. Moreover, they exhibited the highest capacity to support cell migration and to enhance the expression of genes and proteins involved in tissue repair [23]. The diameter and orientation of electrospun fibers were influenced by various electrospinning parameters, including the voltage, collector distance, solution concentration, and collector type. In our experiments, we produced both aligned and crossed fibers using identical electrospinning conditions, resulting in no significant difference in the fiber diameter.

Defining the angle of distribution horizontally to the right as 0°, the fiber orientation was counted: the fiber distribution angle of parallel fibers is 0 ± 4°. A statistical analysis of the cross orthogonal fiber orientation indicated that horizontally distributed fibers had an average angle of 5 ± 4°, while vertically distributed fibers had an angle of 97 ± 5°. Consequently, as depicted in Figure 2f–h, we observed a perpendicular cross-sectional distribution pattern between fibers in the two directions. The diameters and distribution angles of all fibers showed a convergent distribution, indicating good homogeneity in all samples.

Fourier-transform infrared spectroscopy was employed to investigate the surface chemical composition of electrospun membranes. Characteristic absorption peaks were identified in the PU/PLCL composite, including carbonyl (C=O) and imine (C=N) stretching vibrations near 1700 cm^−1^ and 1728 cm^−1^, respectively. A broad absorption band around 3330 cm^−1^, associated with symmetric N–H stretching, confirmed the incorporation of PU components. Moreover, a distinct peak at 1181 cm^−1^ corresponded to C–O stretching vibrations from PLCL chains [32]. Upon blending with PEO, a noticeable blue shift of the 1728 cm^−1^ peak occurred, along with enhanced intensity at 1181 cm^−1^, indicating the successful integration of PEO and modifications to the local chemical environment and bonding interactions (Figure S1).

### 3.2. Cell Spreading and Proliferation

Disease or age-related extracellular matrix (ECM) remodeling often led to the formation of stiff collagen fiber bundles, providing cells with biomechanical cues. In both physiological and pathological contexts, these collagen bundles form distinct topographies. Previous studies have demonstrated that the structure of the ECM influences the cell geometry and participates in the transmission of mechanical and biophysical signals [33]. To precisely and comprehensively investigate the impact of fiber topography on cellular behavior, we developed a “background non-adhesive” fiber patterning platform. This platform effectively eliminates “interfering” cell adhesions, allowing cells to freely explore various shapes in response to well-defined fiber geometries. This provided a chemically stable framework for observing cell adhesion and migration behaviors.

**Figure 3:**
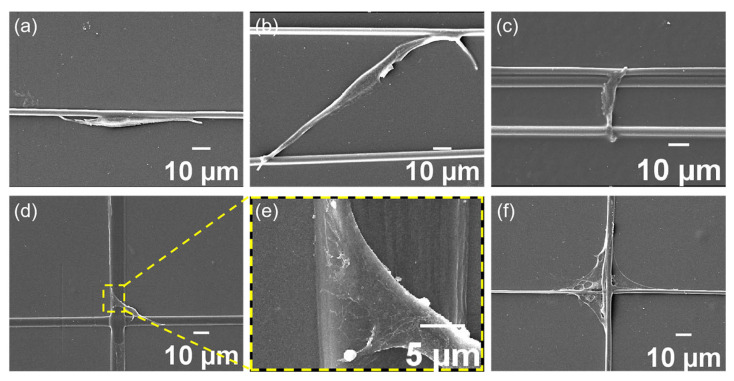
Cell morphology on electrospun fibers with different topologies. (**a**) On the single fiber. (**b**,**c**) Between adjacent fibers. (**d**–**f**) On the crossed fibers.

For aligned fibers, the cell’s long axis aligned with the fiber axis, presenting a significant elongated state and bipolarization forms on both sides of the cell. The topographical guidance provided by electrospun fibers effectively directed cells to spread along the direction of the fibers, as shown in Figure 3a, potentially achieving a balance between cell adhesion restriction and contact guidance on this fiber.

**Figure 4:**
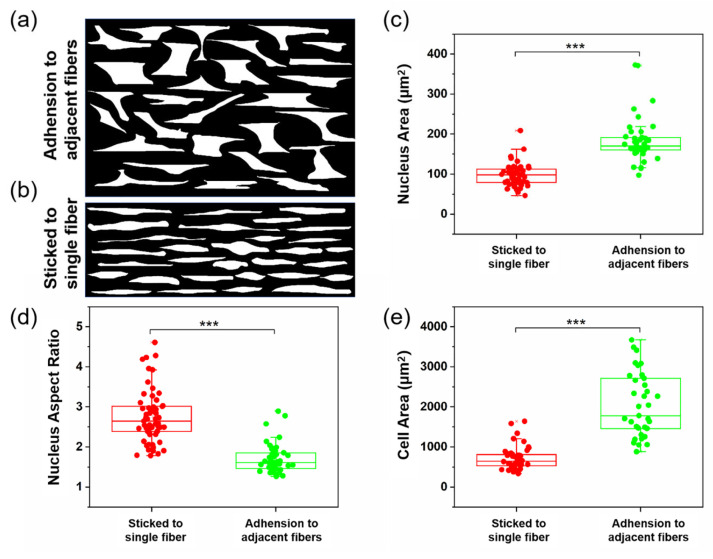
Morphological quantification of cells and nuclei on different topological fibers. (**a**,**b**) Cell spread morphology between the parallel fiber. (**c**) Nuclear area. (**d**) Nuclear aspect ratio. (**e**) Cell area. *** *p* < 0.001.

At the intersections of crossed fibers, the cells usually spread out in stable triangular or rhombic morphologies (Figure 3d–f), which were generated by the contact guidance effect produced by the electrospinning topology. To observe the effect of fiber spacing on cell adhesion behavior, we adjusted the swing frequency of the spinneret to obtain closer fiber spacing, investigating the cell bridging phenomenon on fibers. As shown in Figure 3b,c, cells between fibers with narrower spacing extended pseudopods vertically towards the fibers, attempting to establish connections with multiple fibers during migration, thereby rapidly forming dense arrangements of cross-adhesion between adjacent fibers, ultimately presenting star, spindle, or polygonal cell morphologies (Figure 4a). In another scenario, cells migrated along the original fiber direction throughout the entire tracking process (Figure 4b). Cells forming bridges often exhibited a more rounded nuclear shape, significantly reduced the aspect ratio and orientation, and a markedly increased cell area. In contrast, cells adhering to single fibers demonstrated a higher aspect ratio and orientation (Figure 4c–e). We further compared the spreading area of bridging cells on parallel fibers with that of cells cultured on randomly oriented fibers and found a significantly larger cell area in the former group, as shown in Figure S2. The increased spreading area in bridging cells was associated with more developed actin stress fibers and enhanced mechanotransduction, which facilitated chromatin relaxation in the nucleus and promoted the expression of genes related to cell migration and function. These findings suggested that aligned fibers may provide a more favorable topographical environment for fibroblast activation compared to randomly oriented fibers.

**Figure 5:**
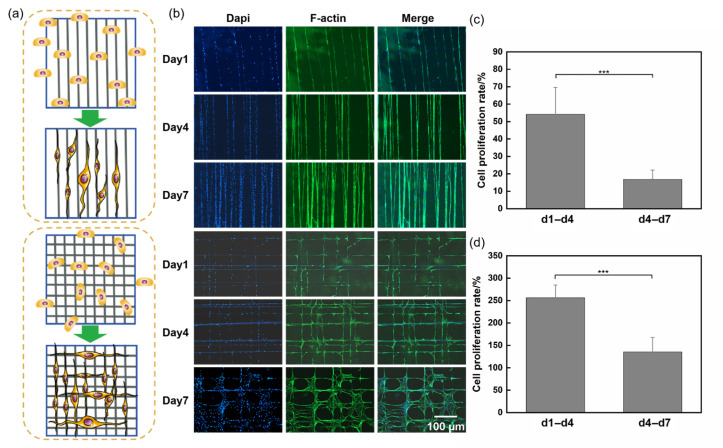
Effects of electrospun fibers with different topologies on the cell proliferation of HSFs. (**a**) Schematic diagram of cell seeding and adhesion on parallel and crossed fibers. (**b**) The results of F-actin and nuclear staining of cells at 1, 4, and 7 day(s) of culture; F-actin was labeled green, and the nucleus was labeled blue. (**c**) Proliferation rate of cells on parallel fibers from day 1 to day 4 and from day 4 to day 7. (**d**) Proliferation rate of cells on crossed fibers from day 1 to day 4 and from day 4 to day 7. The data are expressed as the mean ± standard deviation (n = 3) *** *p* < 0.001.

Cells cultured on fibrous matrices tended to readily align along the surfaces of the fibers and maintained their alignment throughout cultivation, consistent with the observed phenomenon of HSFs aligning along collagen fibers in vivo [34]. Adhesion proteins adsorbed on the fibers from the culture medium played a significant role in enhancing cell attachment and proliferation by facilitating specific interactions between their molecular domains and integrin receptors on the cell surface [35]. To gain deeper insights into the impact of distinct substrate structures on cell proliferation, we conducted staining for cell nuclei and the cytoskeleton to assess cell proliferation on fibers. Figure 5a depicts the progression of cells from initial seeding onto electrospun fibers to subsequent adhesion and spreading. Image J was used to quantify the number of cell nuclei and calculate the proliferation rates from days 1 to 4 and 4 to 7. As shown in Figure 5, the proliferation rate on parallel fibers was 54 ± 20% from day 1 to day 4 and 17 ± 5% from day 4 to day 7. On crossed fibers, the proliferation rate was 256 ± 30% from day 1 to day 4 and 135 ± 30% from day 4 to day 7. The proliferation rate decreased over time on both fiber topographies, likely due to the limited adhesive area available on the fibers. From day 1 to day 4, the larger space available for cell adhesion and proliferation on the fibers resulted in a higher proliferation rate. However, from day 4 to day 7, the non-adhesive background outside the fibers restricted cell proliferation to the fibers, where contact inhibition occurred, leading to a reduced proliferation rate. Additionally, Figure 5b, c showed that the proliferation rate on crossed fibers was higher than on parallel fibers. This may be because the intersections in crossed fibers promote initial cell adhesion and provide a larger area for cell proliferation. Moreover, on the crossed fibers, increased intercellular communication also promoted cell interactions and proliferation [36]. It is shown in Figure 5 that as the number of cells increased, cell adhesion was restricted, prompting pseudopods to extend onto adjacent crossed fibers to form bridges. This bridging behavior significantly alleviated the spatial confinement imposed by single fibers, allowing cells to extend across fiber junctions and thereby promoted broader cell proliferation. In addition, when cells adhered to multiple fibers, this may have led to larger cell protrusions interacting with the fibers, thereby increasing cellular stress and further enhancing cellular function [37].

### 3.3. Cell Migration

**Figure 6:**
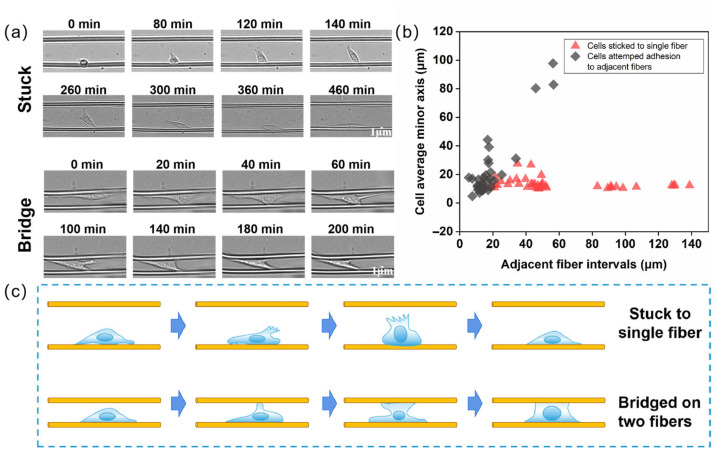
Migration behavior of cells on parallel fibers. (**a**) Time series plot of single cell migration between parallel fibers. (**b**) Relationship between parallel fiber spacing and cell behavior. (**c**) Schematic diagram of the behavior of single cell migrating between parallel fibers.

Using a live-cell workstation, we observed the migration behavior of cells on parallel fibers. As shown in Figure 6a,c, when cells migrate on a single fiber, their major axis was aligned parallel to the fiber axis. The topography provided by the electrospun fibers effectively guided the cells to move along the fiber direction. In some cases, cells may laterally extend their bodies and bridge across multiple fibers (bridging between parallel fibers). In other cases, cells extended pseudopodia to probe the surrounding environment but retracted them and continued migrating in the original direction. Figure 6b further categorizes the cell migration behaviors between parallel fibers, indicating that the distance between fibers (fiber spacing) was a decisive parameter. Therefore, we statistically analyzed the relationship between fiber spacing and cell behavior. The results showed that a fiber spacing of 30–60 μm was a threshold range for distinguishing single-cell behavior. When the distance between fibers was less than 30 μm, cell behavior showed two possibilities: cells either remained on a single fiber or bridged to adjacent fibers. However, when the fiber spacing exceeded 60 μm, cell bridging was not observed; cells remained on the initial fiber or retracted pseudopodia after exploration and returned to the original fiber. This was because the distance between adhesion sites needed to be adequate to generate stress fibers between them, facilitating cell bridging. As shown in the figure, the short axis length of cells is less than 60 μm. The distance between adjacent fibers determined whether cells could deviate from their current migration trajectory, and the size of the cell short axis can be used to measure the cell body’s ability to extend perpendicular to the fiber direction and reach adjacent fibers for migration [38,39].

**Figure 7:**
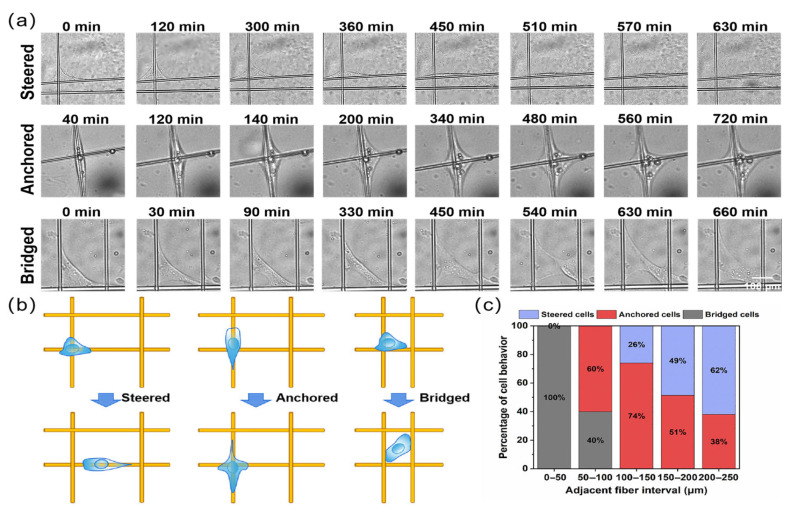
Migration behavior of cells at crossed fibers. (**a**) Time series plot of single cell migration at crossed fibers. (**b**) Schematic diagram of the behavior of single-cell migration at crossed fibers. (**c**) Relationship between the spacing of adjacent fibers at crossed fibers and cell behavior.

Time-lapse imaging was used to observe cell migration on crossed fibers. As shown in Figure 7, most cells were confined to their local grids. This confinement was likely due to cells inevitably encountering fiber intersections within the observed timeframe, where intersections represented local topographical protrusions. When cells encounter these intersections, their protrusions sensed the intersecting fibers, prompting cells to respond and adapt to the underlying structural changes. Additionally, the study found that crossed fibers provided extra degrees of freedom for cell migration compared to parallel fibers. A further investigation into single-cell migration revealed three distinct behaviors at the intersections of crossed fibers, as shown in Figure 7a,b: (1) steering at the intersection, cells were redirected along one of the intersecting fiber paths; (2) anchoring at the intersection, cells remained anchored at the intersection; (3) bridging, cells bridged to adjacent parallel fibers and continued migrating along the two parallel fibers. The more active migratory behavior observed on crossed fibers, as compared to randomly oriented fibers (Figure S3), reflected an enhanced cellular response to the microenvironment. Such behavior implied improved cell–material interactions, including stronger adhesion and traction forces, which are known to promote wound healing and upregulate the expression of functional genes, such as collagen and other ECM-related proteins. Figure 7c presents a statistical analysis of the parallel fiber spacing near intersections. When the fiber spacing was less than 50 μm, only cell bridging was observed. For spacings between 50 and 100 μm, both anchoring and bridging behaviors occurred. When the spacing exceeded 100 μm, cell steering became apparent, and the proportion of steering cells increased with an increasing fiber spacing. This indicated that the distance between adjacent fibers determined the cell behavior at intersections. A fiber spacing of less than 100 μm was a prerequisite for cell bridging, while a spacing greater than 100 μm was necessary for cell steering. In summary, cell migration resulted from the interplay between the external microenvironment and the intrinsic dynamic properties of the cell.

**Figure 8:**
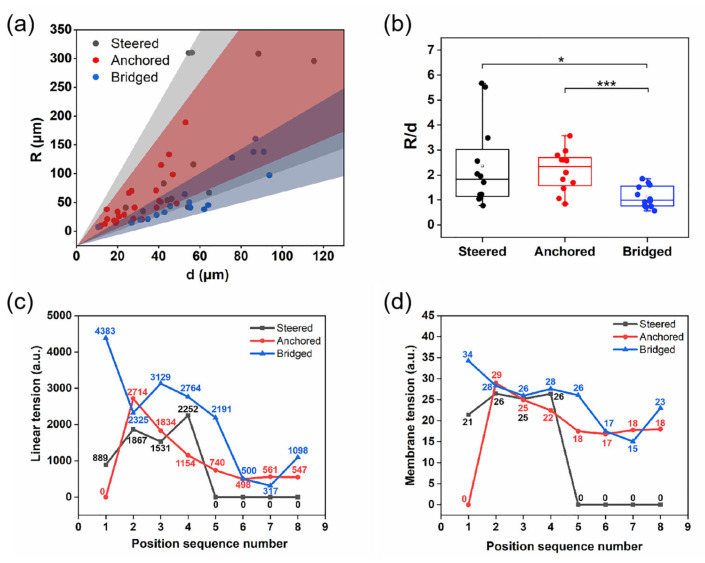
Changes in tension during cell migration at crossed fibers (**a**) Relationship between the radius of curvature *R* and the arc distance *d* of the cell edge. (**b**) Statistical plot of cell edge *R*/*d*. (**c**) Curve of cell edge line tension change during cell migration at the crossing point. (**d**) Curve of cell edge membrane tension change during cell migration at the crossing point. * *p* < 0.05, *** *p* < 0.001.

Using the two-dimensional Laplace law model, the line tension and membrane tension at different time points were calculated for three distinct cell behaviors observed at fiber intersections during cell-crossed fiber interactions. First, the radius *R* of the cell bridging edge and the distance *d* between the two arc endpoints were measured. Since the ratio *R*/*d* can serve as an indicator of the stability of the cell edge, the *R*/*d* values for the three types of cell behaviors were compared, as shown in Figure 8a,b. The results indicated that the *R*/*d* values for bridging cells were significantly lower than those for anchoring and steering cells, suggesting that the cell edges of bridging cells were the least stable. This instability was likely due to the lack of stable support when the cells form bridges between fibers, resulting in mechanically unstable cell edges. Subsequently, the line tension and membrane tension along the cell edges during migration for the three cell behaviors were calculated, as shown in Figure 8c,d. The results showed that, after steering cells completed reorientation and reached a single fiber, both the line tension and membrane tension decreased to nearly zero. Anchoring cells, upon initially sensing the fiber intersection, spread in a diamond-like shape, with their edge line tension and membrane tension reaching peak values; as the cell body progressively extended across the intersection, these tensions gradually decreased. For bridging cells, the edge line tension and membrane tension exhibited a gradual decline during migration between two fibers; however, at the final time point, both tensions rose again, indicating an unstable bridging state and suggesting that further morphological changes are likely to occur.

In summary, regardless of changes in cell morphology, cells consistently tended to adopt configurations associated with reduced edge tension during spreading and migration. This tendency could be attributed to three main factors. First, cell morphology and migration were governed by the mechanical equilibrium, and cells preferentially minimized internal tension and traction forces to reduce overall energy consumption [40]. Second, the cytoskeleton interacted with the extracellular matrix through focal adhesions and integrins, and changes in edge tension influenced the distribution and stability of focal adhesions, thereby affecting cytoskeletal reorganization. A reduction in edge tension promoted a more stable rearrangement of the cytoskeletal structure [41,42]. Third, cells perceived and regulated their surrounding matrix environment through both outside-in and inside-out signaling pathways [43]. When cells detected changes in the external environment, regulatory factors such as focal adhesion proteins and YAP/TAZ responded accordingly, driving the cells to migrate toward configurations associated with lower mechanical tension [44].

## 4. Conclusions

In this investigation, parallel and crossed fibers were fabricated utilizing electrospinning technology, maintaining a uniform fiber diameter and a non-adhesive background to exclude “interfering” cell adhesion in a controlled manner. This approach provided a geometrically controlled environment to explore how cells perceive and interact with the intricate morphology of fibers. The findings revealed the dynamic adaptability of cells to fiber shapes and their responsiveness to adherent and adjacent fibers. Fiber patterns featuring smaller spacing were utilized to examine the impact of bridging between adjacent fibers on cell behavior, offering a partial emulation of the electrospun membrane platform with “interfering” cell adhesion. The ability of cells to modify their migration paths relied on their lateral extension capability to reach neighboring fibers, contingent upon the cell’s minor axis upper limit. A fiber spacing of 30–60 μm served as the threshold range for distinguishing cell behaviors between parallel fibers, while a spacing of 100 μm represented the characteristic dimension that influenced cell behaviors at fiber intersections. Overall, our results demonstrate that the fiber alignment exerts a significant influence on cell morphology and behavior. For researchers aiming to enhance cell proliferation, the use of crossed fibers is recommended. Conversely, if the goal is to induce an elongated cell morphology or promote cell migration, parallel fibers are preferable. These findings provide a useful reference for designing electrospun scaffolds to modulate specific cellular responses through topographical control, providing valuable insights and guidance for designing and fabricating effective fiber biomaterials.

## Figures and Tables

**Table 1:** Table of electrostatic spinning parameters.

Voltage (kV)	Flow Rate (mm/s)	Collector Speed (r/min)	Temperature (°C)	Humidity %	Collection Distance (cm)	Oscillation Range
6	0.0013	3000	25–35	20–30	17	20

## Data Availability

The original contributions presented in this study are included in the article/Supplementary Material. Further inquiries can be directed to the corresponding authors.

## Supplementary Materials

The following supporting information can be downloaded at: https://www.mdpi.com/article/10.3390/ma18143224/s1; Video S1: Steered cells on crossed fibers. Video S2: Anchored cells on crossed fibers. Video S3: Bridged cells on crossed fibers. Figure S1: FTIR spectra of PU/PLCL/PEO composite before and after electrospinning. Figure S2: Cell area statistics of bridging cells on parallel fibers versus cells on randomly oriented fibers. Figure S3: Representative images of HSF migration on electrospun fibers with different architectures.
