# Supplementary material for: Electrospun Parallel, Crossed Fibers for Promoting Cell Adhesion and Migration
Source: Materials (Basel). 2025 Jul 8;18(14):3224. doi: 10.3390/ma18143224 (PMC12300551; doi:10.3390/ma18143224)
Supplement: Supplementary file 1 [file materials-18-03224-s001.zip › materials-3696795-supplementary/Supplementary Material/Supplementary Material.pdf]

Electrospun parallel, crossed fibers for promoting cell adhesion and migration

Xiang Gao [1,2], Jingjun Peng [1], Linjie Huang [1], Xiaoquan Peng [1], Yanjun Cheng [1], Wei Zhang [2,*], Wei Jia [1,*]

1 National Innovation Center for Advanced Medical Devices, National Institute of Advanced Medical Devices, Shenzhen, 518110, China
2 Centre for Translational Medicine Research and Development, Shenzhen Institute of Advanced Technology Chinese Academy of Sciences, Shenzhen, 518055, China
* Correspondence: zhang.wei@siat.ac.cn (W.Z.); wei.jia@nmed.org.cn (W.J.)

Detailed fabrication procedure of randomly oriented fibers:

PLCL and PU were dissolved in a mixture of N, N-Dimethylformamide (DMF) and Trichloromethane (TCM) in a 1:1 ratio. The solution was stirred overnight to obtain a homogeneous electrospinning solution with a concentration of 130 mg/mL. Electrospinning was performed using a high-voltage electrospinning machine (YFSP-T, Tianjin Yunfan Technology Co., ltd., Tianjin, China). The solution was pushed out at a flow rate of 1.2 mL/h using a precision syringe pump. Electrospinning was performed under an applied voltage of 14 kV. The glass substrates were coated with agarose to collect randomly oriented fibers and prevent unnecessary cell adhesion to glass slides.

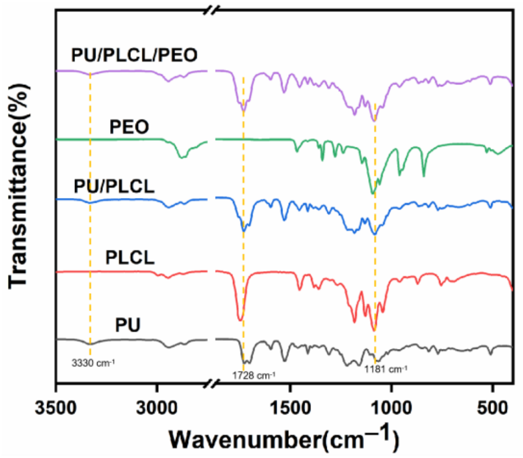

Figure S1. FTIR spectra of PU/PLCL/PEO composite before and after electrospinning.

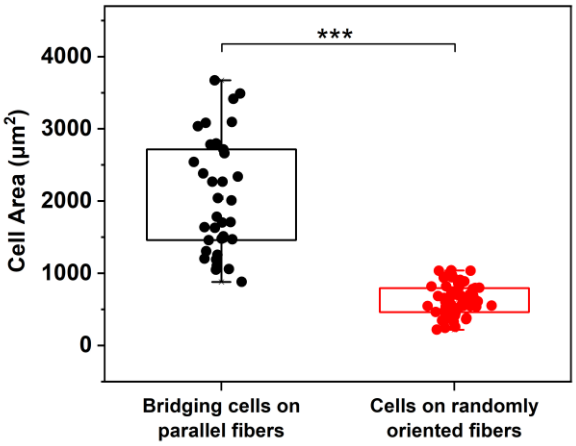

Figure S2. Cell area statistics of bridging cells on parallel fibers versus cells on randomly oriented fibers.

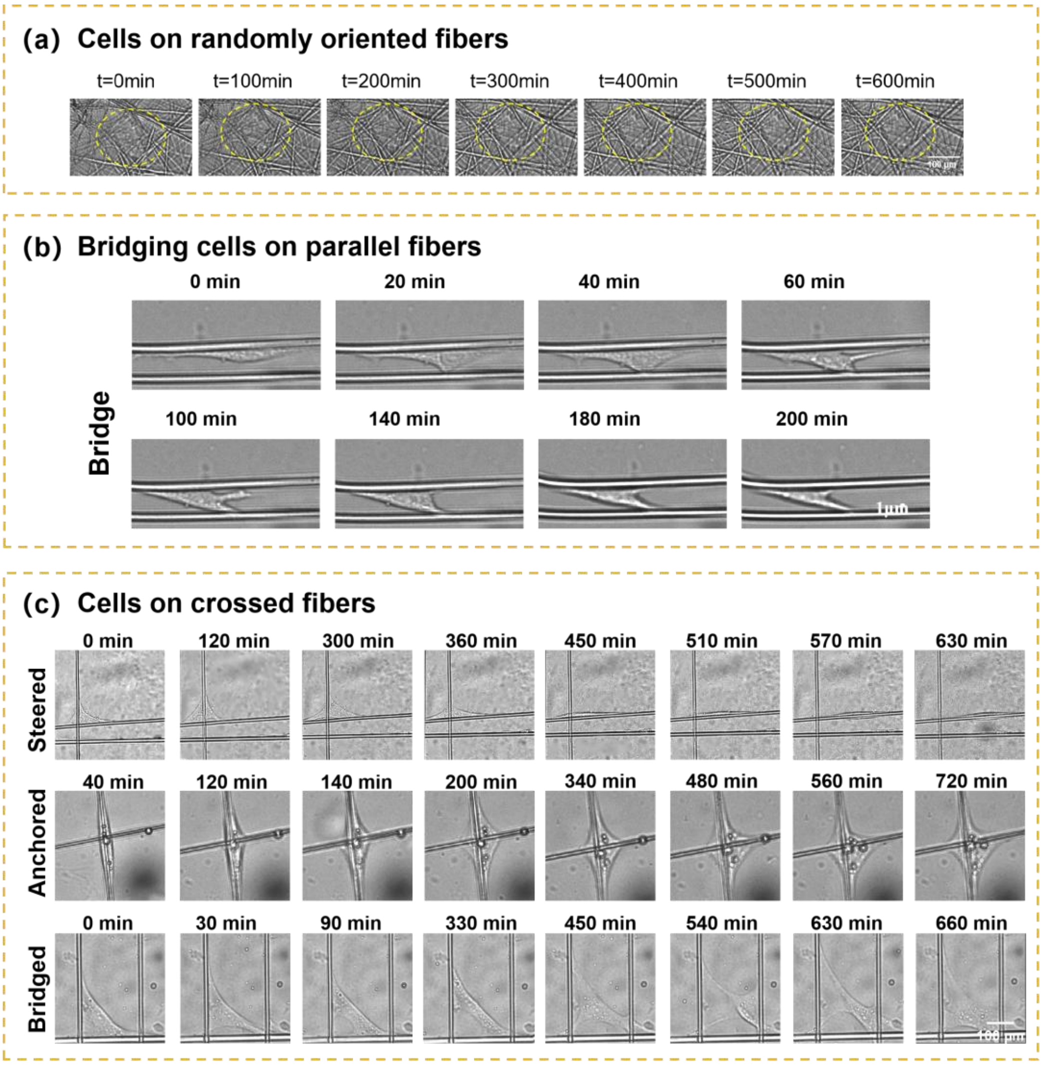

Figure S3. Representative images of HSF migration on electrospun fibers with different architectures.
